# Metformin in Pulmonary Hypertension in Left Heart Disease

**DOI:** 10.3389/fmed.2020.00425

**Published:** 2020-08-19

**Authors:** Vinaya Mulkareddy, Marc A. Simon

**Affiliations:** ^1^Heart and Vascular Institute, University of Pittsburgh Medical Center, Pittsburgh, PA, United States; ^2^Division of Cardiology, Department of Medicine, School of Medicine, University of Pittsburgh, Pittsburgh, PA, United States; ^3^Department of Bioengineering, University of Pittsburgh, Pittsburgh, PA, United States; ^4^Pittsburgh Heart, Lung, Blood and Vascular Medicine Institute, University of Pittsburgh, Pittsburgh, PA, United States; ^5^McGowan Institute for Regenerative Medicine, University of Pittsburgh, Pittsburgh, PA, United States; ^6^Clinical and Translational Science Institute, University of Pittsburgh, Pittsburgh, PA, United States

**Keywords:** pulmonary hypertension, heart failure, therapeutics, metformin, HFpEF

## Abstract

Metformin is ubiquitously used in the management of Type II Diabetes Mellitus (DMII). Over the years, our growing knowledge of its therapeutic potential has broadened its use to the treatment of infertility in polycystic ovarian syndrome, gestational diabetes, and even obesity. Recently, it has been suggested as a novel therapy in cardiovascular disease (CVD). Given that CVD is the leading cause of death in patients with DMII, with ~ 75% dying from a cardiovascular event, the intersection of DMII and CVD provides a unique therapeutic target. In particular, pulmonary hypertension (PH) related to CVD (Group II PH) may be an optimal target for metformin therapy. The objective of this review article is to provide an overview of the pathophysiology of PH related to left heart disease (PH-LHD), outline the proposed pathophysiologic mechanism of insulin resistance in heart failure and PH-LHD, and evaluate the role metformin may have in heart failure and PH-LHD.

## Metformin and Cardiovascular Disease

Several large observational cohort studies have outlined the merits of metformin in CVD in diabetics, including improved outcomes in coronary artery disease (CAD), myocardial infarction (MI), and heart failure (HF). Furthermore, data suggests that metformin may avert the progression and/or development of HF (1). The most current data corroborating this is from supplemental analysis of the EMPA-REG OUTCOME trial, in which patients were randomly assigned to receive empagliflozin 10 mg or 25 mg or placebo daily. Outcomes from this trial include CV death, non-fatal MI or stroke, and hospitalizations. Metformin use, even in the placebo arm of the trial, was associated with improved cardiovascular (CV) outcomes including CV death, HF hospitalizations, major adverse cardiac events, and all-cause mortality (2). The benefit of metformin in CVD is so profound that the American College of Cardiology (ACC) has delineated its benefits in its 2018 ACC consensus, “New Diabetic Drugs to Reduce CV Outcomes” (3). Despite this growing evidence, data on metformin's potential therapeutic role in specific CVD processes is limited.

## Pulmonary Hypertension Due to Cardiovascular Disease

Pulmonary hypertension is a complex, heterogeneous, multisystem syndrome that affects 10–20% of the general population (4). It results from a panvasculopathy that restricts blood flow in the distal pulmonary arteries with eventual maladaptive consequences on the right ventricle. It is a rapidly progressive, debilitating disease process that leads to right ventricular failure, and ultimately death. With its heterogeneous pathophysiology and increasing prevalence, the World Health Organization (WHO) created the Dana Point Clinical Classification, most recently updated by the 6th World Symposium on Pulmonary Hypertension which catalogs the disease into 5 categories based on etiology and pathophysiology (5). The most commonly encountered form of PH is WHO group 2, also known as pulmonary venous hypertension due to left heart disease (4, 6).

PH is estimated to affect up to 35% of patients with left heart disease (LHD). PH-LHD is defined as a mean pulmonary artery pressure of > 20 mmHg with a concomitant pulmonary artery wedge pressure of > 15 mmHg (7). As we uncover more about PH-LHD, it is becoming increasingly apparent that the pathophysiologic aberrations and the phenotypic variations are highly dependent on the underlying cardiovascular disease process, e.g., heart failure with reduced ejection fraction (HFrEF) vs. heart failure with preserved ejection fraction (HFpEF) (4, 7, 8).

HFrEF is characterized by signs and symptoms of HF in the setting of abnormal left ventricle (LV) systolic function. It is defined by a LV ejection fraction (LVEF) of ≤ 40%, progressive LV dilation, eccentric remodeling, reduced LV systolic elastance, and increased arterial elastance. Regardless of the etiology, there are a number of beneficial therapeutic interventions available including beta-adrenergic receptor blockers, aldosterone antagonists, ace-inhibitors, angiotensin receptor blockers, hydralazine and isosorbide dinitrate, intracardiac defibrillators, and cardiac resynchronization therapy. Unfortunately, the benefits of these therapies do not translate to PH-HFrEF, as HFrEF patients with PH continue to have worse outcomes than those without (9–12).

In contrast, HFpEF is a complex, heterogeneous, systemic syndrome characterized by signs, and symptoms of HF in the setting of a normal left ventricular ejection fraction. A number of cardiac and extracardiac aberrations and comorbidities result in HFpEF's heterogeneity and phenotypic variation (13, 14). Unlike in HFrEF, there are a paucity of beneficial therapeutic interventions available in HFpEF raising the question of whether targeting specific HFpEF sub-phenotypes may lead to, as of yet, elusive successful therapies. One such unique phenotype is pulmonary hypertension (PH).

PH is prevalent in ~ 60–70% of HFrEF patients and 80% of HFpEF patients and is also associated with a higher morbidity and mortality (15). The mechanism and pathophysiology of PH in HF, as alluded to previously, is complex and incompletely understood. Initially elevated LV end-diastolic pressure and secondary mitral regurgitation leads to chronically elevated left atrial pressure and increased pulmonary artery wedge pressure. This contributes to both pulmonary vasoconstriction and alveolar capillary stress failure. Eventually over time there is increased pulmonary vascular remodeling, endothelial dysfunction, intimal fibrosis, and venous hypertension. The venous congestion from left atrial hypertension in conjunction with reactive arterial vasoconstriction and remodeling is referred to as combined pre- and post-capillary PH, defined hemodynamically as a mean pulmonary artery pressure >20 mmHg with a pulmonary artery wedge pressure > 15 mmHg and pulmonary vascular resistance ≥ 3 Woods units (5, 7). Other pathophysiologic mechanisms have also been implicated in PH-LHD as well-including intrinsic myocyte stiffness, extracellular matrix alterations, microvascular dysfunction, inflammation, and metabolic dysfunction (7, 16). Given the lack of available therapies, investigating alternative pathophysiologic mechanisms such as metabolic syndrome or insulin resistance as novel therapeutic targets is paramount.

## Metabolic Syndrome, Insulin Resistance, PH-LHD

Heart failure (HF) is a heterogeneous syndrome with regards to demographics, underlying etiology, disease course, and prognosis. To better categorize and risk stratify this complex syndrome, different schema have been developed including the American Heart Association (AHA) stages as well as the New York Heart Association classification (17). Over the last several years, greater efforts have been made to sub-phenotype HF in order to improve risk stratification and facilitate therapeutic interventions. This stratification has led to a greater awareness of the role of metabolic syndrome in HF as well as PH-LHD.

DM is closely associated with HF. A meta-analysis of HF clinical trial populations uncovered that the prevalence of DMII in HF patients approximates 30% (18). This increases to 40% in African Americans. Additionally, patients with concomitant HF and DM have increased morbidity and mortality. In the Candesartan in Heart Failure Assessment of Reduction in Mortality and Morbidity trial (CHARM), DM was an independent predictor of cardiovascular death and hospitalization in patients with HFpEF (19). More recently, DMII has been linked as an independent risk factor in the development of HF as publicized by data derived from the Framingham Risk Study. Both men and women with DMII were 5x more likely to develop HF even after taking into account incidence of CAD (20). Furthermore, 60% of HF patients with normal fasting glucose and insulin levels were found to have abnormal responses to oral glucose tolerance testing—implicating insulin resistance as an underlying pathophysiologic contributor to HF (20). Given this, traditional and novel glycemic control agents are being increasingly studied as a therapeutic agent in the management of HF. A list of such agents is summarized in Table 1.

**Table 1:** Antiglycemic agents and cardiovascular outcomes.

**Agent**	**Trial**	**CV death**	**All-cause death**	**HF hospitalizations**
Empagliflozin	EMPA-REG (2)	0.62 (0.49–0.77)	0.68 (0.57–0.82)	0.65 (0.50–0.85)
Canagliflozin	CANVAS (21)	0.87 (0.72–1.06)	0.87 (0.74–1.01)	0.67 (0.52–0.87)
Saxagliptin	SAVOR-TIMI53 (22)	1.03 (0.87–1.22)	1.11 (0.96–1.27)	1.27 (1.07–1.51)
Sitagliptin	TECOS (23)	1.03 (0.89–1.19)	1.01 (0.90–1.14)	1.00 (0.83–1.20)
Liraglutide	LEADER (24)	0.78 (0.66–0.93)	0.85 (0.74–0.97)	0.87 (0.73–1.05)
Semaglutide	SUSTAIN-6 (25)	0.98 (0.65–1.48)	1.05 (0.74–1.50)	1.11 (0.77–1.61)
Exenatide	EXSCEL (26)	0.88 (0.76–1.2)	0.86 (0.77–0.97)	0.94 (0.78–1.13)

*All numerical values including cardiovascular death, all cause death, and heart failure hospitalizations are presented as hazard ratios with the 95% confidence interval. CV indicates cardiovascular; HF indicates heart failure*.

It is hypothesized that insulin resistance directly affects myocardial structure and function through lipotoxicity, free fatty acid oxidation, oxidative stress, impaired nitric oxide bioavailability, and mitochondrial dysfunction and fibrosis (27–31). Impaired insulin signaling diminishes myocardial glucose extraction and uptake resulting in increased fatty acid oxidation (20). Free fatty oxidation in turn leads to accumulation of lipid storage molecules including triglycerides, ceramide, and diacylglycerol in cardiomyocytes. Ceramide induces apoptosis and impaired mitochondrial function, which eventually results in ventricular dysfunction and remodeling. The histologic outcome is similar to what is observed in pressure overload, volume overload, or myocardial infarction (20).

The role of insulin resistance in HF and PH-LHD has been well-documented in preclinical models. For example, altered glucose metabolism involving downregulation of 5′adenosine monophosphate-activated protein kinase (AMPK) has been implicated in the pathogenesis of PH-LHD. Mice exposed to a high fat diet and L-NAME (NO synthase suppression) to induce metabolic syndrome and hypertension were found to have many of the hallmarks of HFpEF including exercised intolerance, lung congestion, LV hypertrophy, cardiac fibrosis, reduced myocardial capillary density, increased left ventricular filling pressure, reduced LV global longitudinal strain despite preserved LV ejection fraction (EF), and decreased contraction velocity with impaired relaxation (32).

In another preclinical model, metabolic syndrome, and left ventricular dysfunction were induced in mice through supracoronary aortic banding and a high fat diet supplemented by olanzapine. In this model, metabolic syndrome exacerbated group 2 PH via vascular remodeling. There was increased macrophage accumulation, interleukin 6 (IL-6), and signal transducer and activator of transcription 3 (STAT3). These findings were associated with increased proliferation of smooth muscle cells in pulmonary arteries, remodeling of distal pulmonary arteries, and PH (33).

Metabolic changes resulting from abnormal glucose handling and insulin resistance were found to precede the structural and functional aberrations in cardiac muscle. This was demonstrated through glucose-6-phosphate (G6P) via mammalian target of rapamycin (mTOR) and endoplasmic reticulum stress in *ex vivo* animal models, *in vivo* animal models, and in humans with HF (34). While under stress such as increased inotropic demand, cardiac myocytes undergo both metabolic, and structural remodeling. Metabolically, carbohydrates become the main energy source. Structurally, cardiac hypertrophy has been linked to increased activity of mTOR, a regulator myocardial protein synthesis (35). Rats pretreated with various energy sources including glucose, non-carbohydrate energy substrate, or glucose analog underwent *ex vivo* analysis of cardiac muscle exposed to high workloads. There was increased G6P accumulation, mTOR activity, endoplasm reticulum stress, and impaired contractility regardless of energy source. This was not appreciated in rats pretreated with rapamycin (an mTOR inhibitor) or metformin (an AMPK activator). This was re-demonstrated with *in vivo* rat models. Rats exposed to aortic constriction had increased glucose tracer analog uptake and contractile dysfunction utilizing micro-PET imaging (34). These changes preceded LV dilation. Furthermore, humans with heart failure were found to have decreased G6P and endoplasmic reticulum stress markers once the heart was mechanically unloaded utilizing left ventricular assist devices (34). Pathophysiologically, it is hypothesized that increasing workload surpasses cardiomyocyte glucose uptake capability and oxidative capacity. This results in G6P accumulation, a crucial mediator in load-induced mTOR activation. mTOR impairs cardiac power and induces ER stress leading to contractile dysfunction and hypertrophy. Rapamycin, metformin, and mechanical unloading reversed this metabolic cascade (34).

### Impact

HF is a worldwide epidemic, affecting ~ 6 million people in the United States and 23 million people worldwide (36). Approximately 1 in 5 adults over the age of 40 will develop HF and survival estimates are as low as 70% at 1 year and 50% at 5 years. Furthermore, it costs the United States roughly 30 billion dollars per year. This is expected to more than double by 2030 (36). Morbidity and mortality of HF patients with concomitant PH is significantly higher (10, 11, 37–40). It is also apparent that there is continuum of risk with even borderline PH associated with higher mortality and morbidity (41). Unfortunately, once HF patients develop PH there are no therapies to reverse or even cease progression.

Thus far, treatment of PH-LHD is geared toward managing volume status with diuretics and dietary modifications, implementing goal directed medical therapy for left ventricular dysfunction, and treating comorbidities such as systemic hypertension. Despite medical optimization with the aforementioned therapies, elevated pulmonary pressures persist, and often worsen. Traditional PAH therapies have been studied, but unfortunately with generally negative outcomes. Endothelin Receptor Antagonists (ERA) trials such as ENABLE (Endothelin Antagonist Bosentan for Lowering Cardiac Events in Heart Failure) reported no benefit in regards to mortality and in fact found increased heart failure exacerbations and heart failure hospitalizations secondary to fluid retention (42). Prostacyclins have been associated with improved cardiac output and decreased pulmonary vascular resistance but have had a strong trend toward higher mortality (43). Phosphodiesterase-5 inhibitors in clinical trials have had disappointing results. For example, RELAX (Phosphodiesterase-5 Inhibition to Improve Clinical Status and Exercise Capacity in Diastolic Heart Failure) showed no significant improvement in clinical outcomes (44). Uncovering novel therapies to prevent the development of PH-LHD from HF or even halt the progression of PH-LHD is paramount given the growing public health concern that HF and PH-LHD poses. Given the association of insulin resistance with HF and PH-LHD, metformin is of high interest as a novel treatment.

## Preclinical Models: Metformin's Mechanistic Evidence

**Figure 1:**
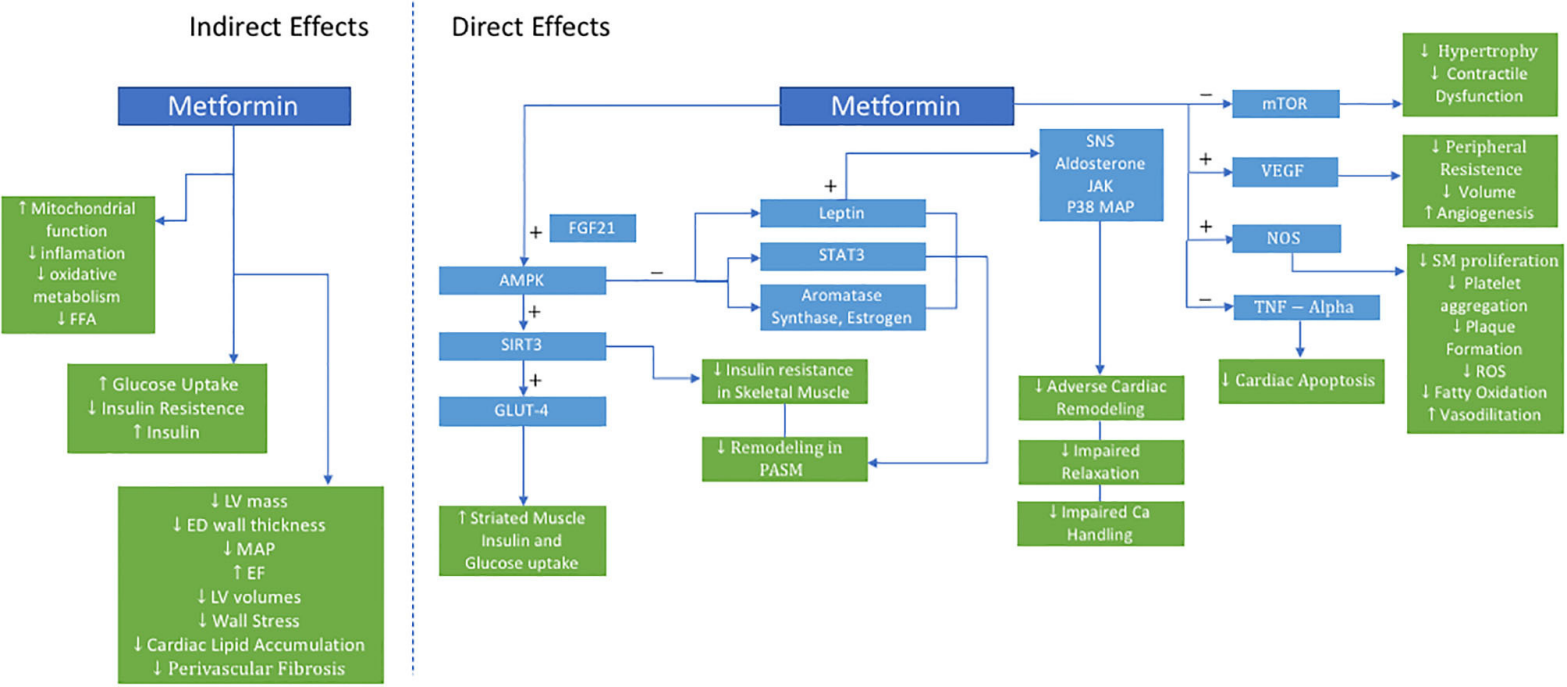
Proposed Actions of Metformin in PH-HFpEF. AMPK indicates 5′-AMP-activated protein kinase; Ca, calcium; ED, end-diastolic; EF, ejection fraction; FGF21, fibroblast growth factor 21; FFA, free fatty acid; LV, left ventricle; GLUT4, glucose transporter type 4; JAK, janus kinase; LV, left ventricle; MAP, mean arterial pressure; mTOR, mammalian target of rapamycin; NOS, nitric oxide synthase; P38 MAP, P38 mitogen-activated protein; PASM, pulmonary artery smooth muscle; ROS, reactive oxygen species; SIRT3, sirtuin-3; SM, smooth muscle; SNS, sympathetic nervous system; STAT3, signal transducer and activator of transcription 3; TNF, tumor necrosis factor; VEGF, vascular endothelial growth factor.

Mechanistically, metformin exerts its benefit on the CV system in a pleotropic manner (Figure 1). It is hypothesized that these pleotropic effects are due to its role in directly activating AMPK, which is a cellular energy sensor that is intricately involved in numerous metabolic processes not limited to liver and skeletal muscle glucose metabolism (45).

In skeletal muscle, metformin enhances glucose-mediated glucose uptake resulting in improved insulin sensitivity, oxidative metabolism, mitochondrial function (46). Dysregulation of AMPK and its downstream signaling network have both been implicated in metabolic syndrome, HF, and PH. This may be further mediated upstream via sirtuin-3 (SIRT3), a mitochondrial deacetylase. When downregulated, SIRT3 contributes to the development of insulin resistance, and eventually diabetes. Furthermore, SIRT3-AMPK downregulation in pulmonary artery smooth muscle cells is associated with the development of PH in leptin deficient, obese, diabetic rats with metabolic syndrome, and was reversed with metformin (47). Metformin restored SIRT 3 in skeletal muscles, improved insulin sensitivity, and was associated with decreased pulmonary pressures (47).

Metformin upregulates the SIRT3-AMPK-Glut4 pathway. Glucose transporter type 4 (Glut 4), an insulin-regulated glucose transporter in adipose tissue and striated muscle, improves insulin action and glucose uptake. Myokines, such as fibroblast growth factor 21 (FGF21) which regulates simple sugar intake, may be a critical link in crosstalk between skeletal muscle metabolism and pulmonary vascular smooth muscle cells, further activating AMPK, and preventing pulmonary vascular remodeling. Additional mechanisms of action of metformin on vascular remodeling include AMPK-mediated inhibition of estrogen and aromatase synthesis, inhibition of leptin secretion, and downstream STAT3 activation—all crucial mediators of group 2 PH (48).

Additionally, metformin augments weight loss, directly treating the adverse effects of obesity (19). Over 80% of HF patients in clinical trials and epidemiologic cohorts are overweight or obese. Mechanistically, obesity is hypothesized to have pleiotropic effects on HF and PH-LHD as a result of abnormal endocrine, cellular, and cardiometabolic signaling. Important mediators of this include adipokines including leptin and adiponectin. Leptin directly affects the sympathetic nervous system, aldosterone receptor signaling, janus kinase (JAK) signal transducer, STAT proteins, and p38 mitogen-activated protein kinase, all of which have been implicated in deleterious cardiac remodeling, impaired calcium handling, and impaired relaxation. Adiponectin decreases with obesity and is linked to increasing inflammation, oxidative stress, as well as adverse cardiac remodeling (49). While the effect of obesity on HF has been previously described, obesity's impact on the pulmonary vasculature in the PH-LHD phenotype is still unknown.

Metformin has also been shown to have a beneficial role in chronic systemic hypertension preventing the development and progression of hypertensive LHD. Spontaneously hypertensive rats were noted to have significant myocardial metabolic changes even prior to developing cardiac dysfunction. Metformin treatment in this model lowered mean arterial pressure, lowered glucose uptake, improved ejection fraction, and decreased left ventricular mass and end-diastolic wall thickness. Furthermore, free fatty acid levels and mTOR activity were reduced as well (50). In another model, spontaneous hypertensive, insulin-resistant rats (SHHF) treated with metformin had decreased LV volumes, wall stress, cardiac lipid accumulation, and perivascular fibrosis—all markers of LV remodeling. Additional improvements were seen in indices systolic and diastolic ventricular function. Metformin activated AMPK, vascular endothelial growth factor, and nitric oxide synthase, while reducing myocyte apoptosis and tumor necrosis factor-alpha expression—all have which been implicated in HF pathogenesis. These metabolic, molecular, and structural changes translated to functional cardiomyocyte improvements and prevention of HF (51).

## Clinical Trials

Will preclinical, observational, and *post-hoc* data translate into distinct measurable benefit for HF or PH-LHD? This has yet to be determined, but there is certainly reasonable evidence to support clinical trials (52). In a compelling pilot study recently completed, Mohan et al. (53) assessed the therapeutic benefit of metformin on LV hypertrophy in 68 patients with coronary artery disease without diabetes. They showed metformin decreased LV mass, systolic blood pressure, body weight, and oxidative stress as measured by thiobarbituric acid reactive substances in patients with preserved LVEF (53). Another trial in 62 non-diabetic insulin resistant HFrEF patients found improvements in functional class and minute ventilation/carbon dioxide production (VE/VCO2 slope) (54). We are currently pursuing a prospective phase II clinical trial evaluating the therapeutic efficacy of metformin in PH-HFpEF (Clinicaltrials.gov identifier NCT03629340) which will evaluate exercise hemodynamics, functional capacity, skeletal muscle signaling, and insulin sensitivity.

## Metformin in PAH and HFPEF

Briefly, although beyond the scope of this review, the therapeutic role of metformin is also being investigated in both pulmonary arterial hypertension (PAH) and HFpEF. There is a growing body of evidence that PAH is associated with a number of systemic metabolic derangements including metabolic syndrome, insulin resistance, and glucose intolerance. Alterations in aerobic glycolysis, tricarboxyclic acid metabolism, and fatty oxidation have all been implicated in the pathophysiology. Given these findings, there is at least one currently ongoing clinical trial (NCT01884051) looking at the role of metformin in PAH (55). While in HFpEF there is significant clinical heterogeneity, there is increasing evidence illustrating metformin's therapeutic benefit across the varying phenotypes. Halabi et al. (56) recently published a systematic review and meta regression analysis analyzing the role of metformin in HFpEF. Metformin reduced mortality and morbidity in HFpEF patients even after adjustment for other HF and glycemic control therapies including beta blockers, angiotensin converting enzyme inhibitors, and insulin (56).

## Author Contributions

All authors contributed to all aspects of the manuscript.

## Conflict of Interest

MS: Research support from Novartis, Aadi. Consultancy fees from Complexa, Actelion, United Therapeutics, Acceleron. The remaining author declares that the research was conducted in the absence of any commercial or financial relationships that could be construed as a potential conflict of interest. The reviewer BM has declared a past co-authorship with one of the author MS to the handling Editor.
